# Selective Induction of Cell Death in Melanoma Cell Lines through Targeting of Mcl-1 and A1

**DOI:** 10.1371/journal.pone.0030821

**Published:** 2012-01-24

**Authors:** Daniela Senft, Carola Berking, Saskia A. Graf, Claudia Kammerbauer, Thomas Ruzicka, Robert Besch

**Affiliations:** Department of Dermatology and Allergology, Ludwig-Maximilian University, Munich, Germany; Universitat de Lleida – IRBLLEIDA, Spain

## Abstract

Melanoma is an often fatal form of skin cancer which is remarkably resistant against radio- and chemotherapy. Even new strategies that target RAS/RAF signaling and display unprecedented efficacy are characterized by resistance mechanisms. The targeting of survival pathways would be an attractive alternative strategy, if tumor-specific cell death can be achieved. Bcl-2 proteins play a central role in regulating survival of tumor cells. In this study, we systematically investigated the relevance of antiapoptotic Bcl-2 proteins, i.e., Bcl-2, Bcl-xL, Bcl-w, Mcl-1, and A1, in melanoma cell lines and non-malignant cells using RNAi. We found that melanoma cells required the presence of specific antiapoptotic Bcl-2 proteins: Inhibition of Mcl-1 and A1 strongly induced cell death in some melanoma cell lines, whereas non-malignant cells, i.e., primary human fibroblasts or keratinocytes were not affected. This specific sensitivity of melanoma cells was further enhanced by the combined inhibition of Mcl-1 and A1 and resulted in 60% to 80% cell death in all melanoma cell lines tested. This treatment was successfully combined with chemotherapy, which killed a substantial proportion of cells that survived Mcl-1 and A1 inhibition. Together, these results identify antiapoptotic proteins on which specifically melanoma cells rely on and, thus, provide a basis for the development of new Bcl-2 protein-targeting therapies.

## Introduction

Melanoma is one of the deadliest types of skin cancer with strongly rising incidence. Due to its therapy resistance in advanced stages, melanoma is the skin cancer with the highest mortality. Fewer than 20% of melanoma patients respond to chemotherapy, which does not prolong the survival of late stage melanoma patients [1], [2]. Recent studies indicate that targeting the MAP kinase signaling pathway, which is commonly activated in melanoma, represents an important new therapeutic approach. Inhibitors that specifically target the most common V600E mutant form of BRAF displayed a remarkable tumor response even in advanced melanoma [3]. However, there is evidence that melanoma cells can become resistant to RAF inhibition [4], [5].

The targeted activation of apoptotic pathways may be an alternative antitumor strategy and may be valuable to overcome de novo or acquired resistance to conventional chemotherapy or MAP kinase inhibition. Apoptosis can be initiated via two pathways, the mitochondrial and the death receptor-mediated pathway [6]. Critical for regulation of the mitochondrial apoptosis pathway are molecules of the Bcl-2 family [7]. This family consists of antiapoptotic proteins like Bcl-2, Bcl-xL, Bcl-w, Mcl-1, and A1, and proapoptotic proteins like Bax, Bak, and the BH3-only subgroup. A shift in the balance of antiapoptotic Bcl-2 proteins and proapoptotic BH3-only proteins results in activation of Bax and Bak at the outer mitochondrial membrane, which leads to permeabilization of the outer mitochondrial membrane and to the release of cytochrome c into the cytosol. Cytosolic cytochrome c leads to the formation of complexes, called apoptosomes, which results in caspase activation and cell death. The molecular mechanisms by which antiapoptotic Bcl-2 proteins and proapoptotic BH3-only proteins regulate Bax or Bak activation is not entirely clear [8], [9].

The group of antiapoptotic Bcl-2 proteins consists of five members, i.e., Bcl-2, Bcl-xL, Bcl-w, Mcl-1, and A1. The expression level and relevance for survival of each antiapoptotic member varies between different cell lineages. Antiapoptotic proteins can promote survival and, accordingly, the expression or the activity of antiapoptotic proteins can be increased in cancer. In addition, cellular stress in tumors, e.g., generated by genomic alterations, exaggerated proliferation, or insufficient nutrition can result in the requirement of antiapoptotic proteins for tumor cell survival. This situation, termed synthetic lethality, can make tumor cells vulnerable and offers the opportunity for therapeutic intervention [10], [11]. Indeed, several synthetic inhibitors, the so-called BH3 mimetics, have been developed that counteract the activity of antiapoptotic proteins. These molecules inhibit certain members of the antiapoptotic Bcl-2 subgroup and, therefore, display different activity in each cell type [12].

In this study, we systematically investigated the relevance of antiapoptotic Bcl-2 proteins in melanoma cell lines utilizing RNA interference. In addition, primary human fibroblasts from skin were studied in order to identify those antiapoptotic Bcl-2 proteins whose loss specifically affects melanoma cells while sparing non-malignant cells. It was found that melanoma cell lines – in contrast to non-malignant fibroblasts - required specific antiapoptotic Bcl-2 proteins for survival. Inhibition of Mcl-1 and A1 resulted in cell death of melanoma cell lines and targeting both antiapoptotic Bcl-2 proteins simultaneously enhanced cell death to 80% whereas non-malignant cells remained unaffected. In addition, cell death induction could be further increased by exposure to chemotherapeutic drugs.

## Results

### Expression of antiapoptotic Bcl-2 proteins in melanoma

**Figure 1:**
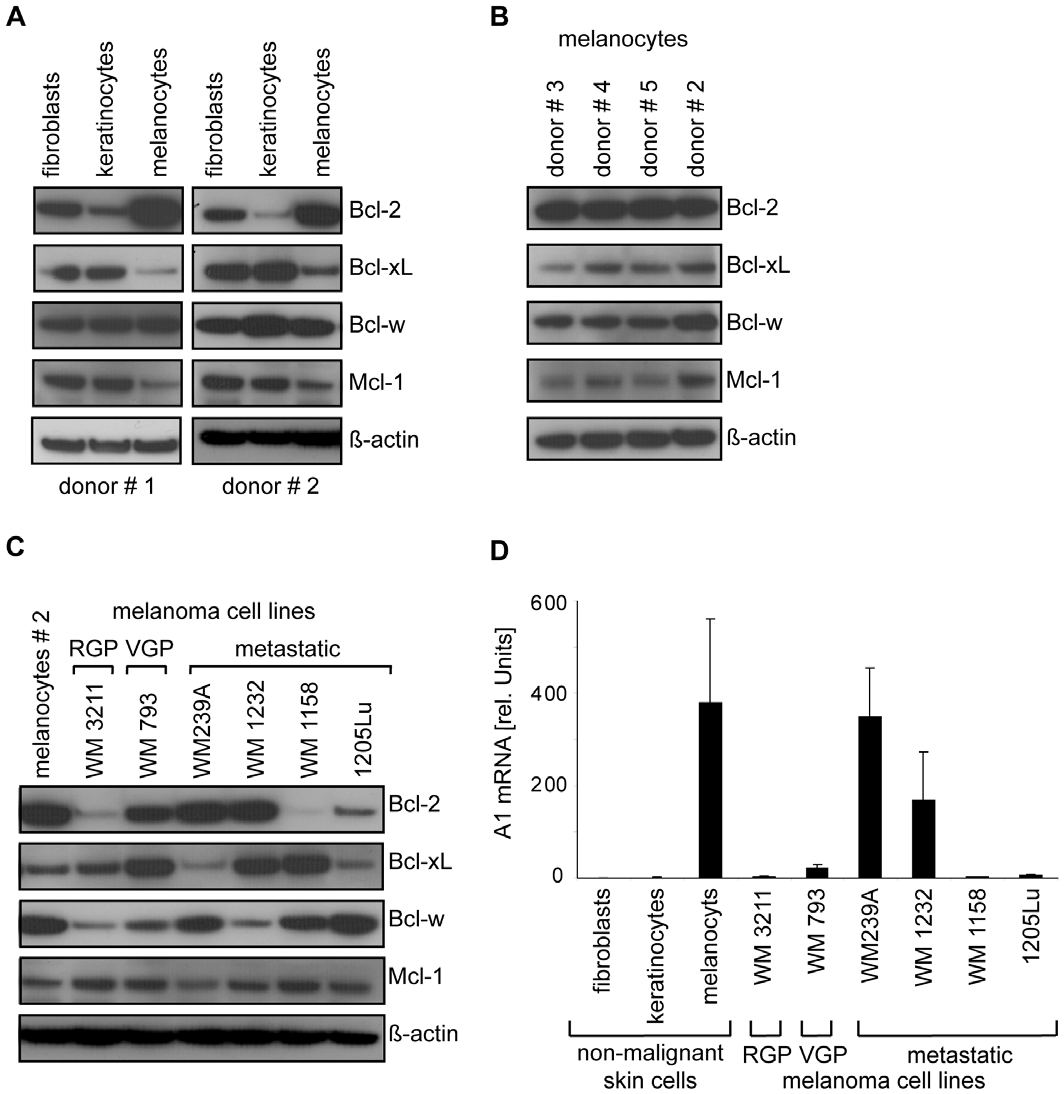
Expression of antiapoptotic Bcl-2 proteins in non-malignant skin cells and melanoma cell lines. (A) Expression of Bcl-2, Bcl-xL, Bcl-w, and Mcl-1 protein in primary human fibroblasts, keratinocytes, and melanocytes was analyzed by immunoblotting. For each cell type, 2 donors are shown. (B) Expression of Bcl-2, Bcl-xL, Bcl-w and Mcl-1 protein in melanocytes isolated from 4 donors. (C) Expression of Bcl-2, Bcl-xL, Bcl-w and Mcl-1 protein in melanoma cell lines of different progression levels. Blot is representative for 2 independent experiments. (D) Levels of A1 mRNA were measured by quantitative RT-PCR. Mean +/− SD of 3 donors is shown for non-malignant cells, mean +/− SD of 3 cell passages is shown for melanoma cell lines. β-actin served as loading control in immunoblots. The same protein sample of melanocytes from donor #2 was loaded on all gels as a reference to allow the comparison of expression levels among the immunoblots shown in A, B, and C. RGP: radial-growth phase (non-invasive); VGP: vertical growth phase (invasive).

Expression levels of antiapoptotic Bcl-2 proteins were analyzed by immunoblotting. Because sensitivity of commercial A1 antibodies was found to be not sufficient for detection, expression levels of A1 were analyzed on RNA level. Compared to primary human keratinocytes and fibroblasts, primary human melanocytes expressed lower levels of Bcl-xL and Mcl-1 whereas levels of Bcl-2 and A1 were higher (Figure 1A and 1D). This melanocytic expression signature was confirmed in several donors (Figure 1B). Next, melanoma cell lines of non-invasive, invasive, and metastatic origin were analyzed. The expression pattern was clearly altered to that of melanocytes (Figure 1C and 1D). Some melanoma cell lines, e.g., WM793, WM1232, or WM1158, expressed high levels of Bcl-xL suggesting a survival advantage, however this was not consistent throughout the panel and low Bcl-xL levels were observed as well (e.g., in 1205Lu). In addition, antiapoptotic Bcl-2 proteins like Bcl-2, Bcl-w, or A1, which were strongly expressed in melanocytes, were only expressed at low levels in some melanoma cell lines, such as WM3211, WM1158, and WM1232.

These results suggest that melanocytes express a characteristic expression pattern of antiapoptotic Bcl-2 proteins, which is clearly altered in melanoma cells. In contrast to melanocytes, the expression profile in melanoma cells was inconsistent among different cell lines. Overall, no general increase in the expression of antiapoptotic Bcl-2 proteins was observed in melanoma cell lines, which would point towards a pro-survival role.

### Inhibition of Mcl-1 or A1 leads to cell death specifically in melanoma cell lines

**Figure 2:**
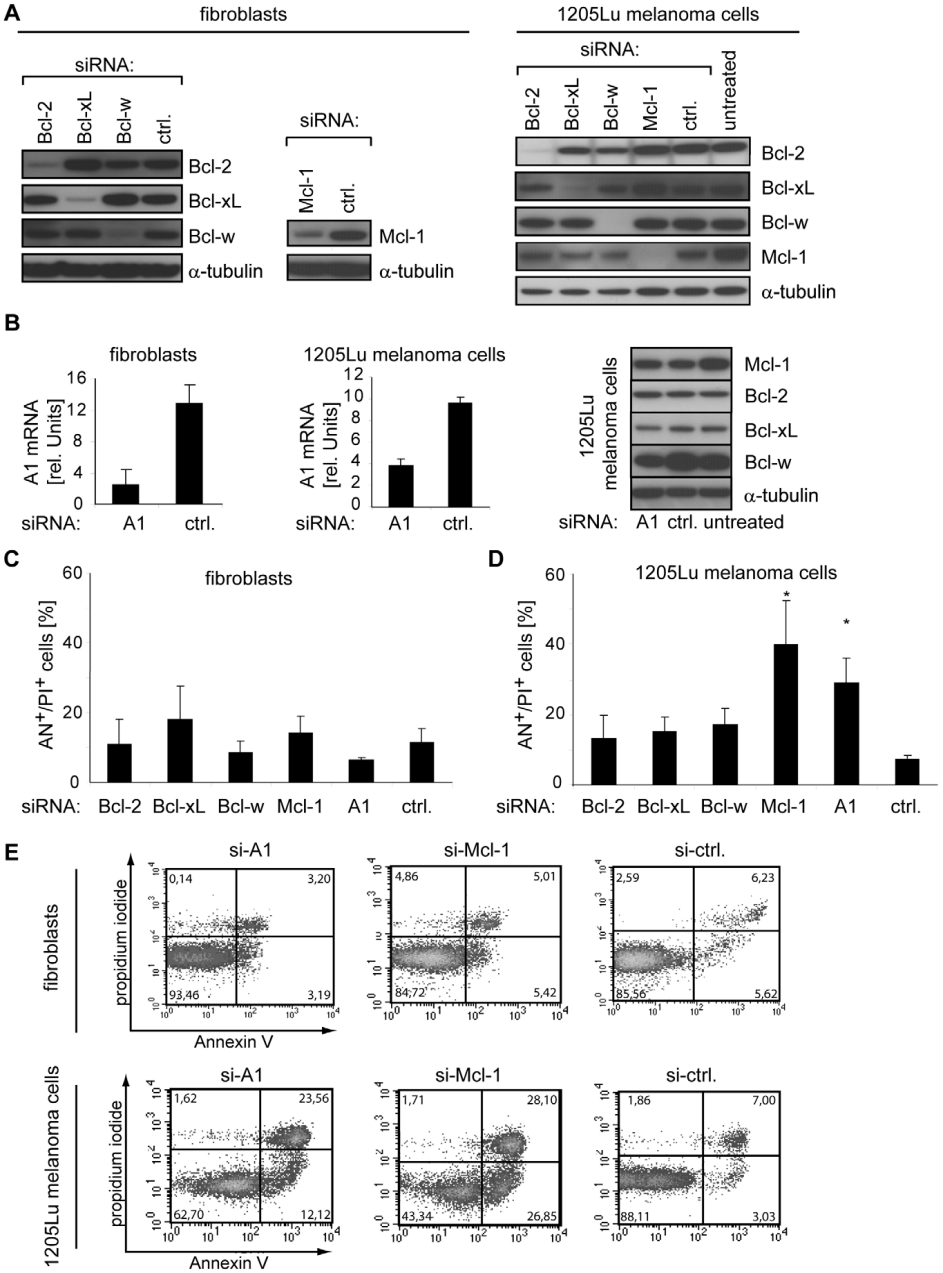
Inhibition of Mcl-1 or A1 leads to melanoma-specific cell death. (A) Primary human fibroblasts and 1205Lu melanoma cells were treated with the indicated siRNAs and expression of Bcl-2, Bcl-xL, Bcl-w, and Mcl-1 was analyzed after 48 hours by immunoblotting. (B) A1 mRNA was measured by quantitative RT-PCR in cells treated with A1-specific- or control siRNA for 48 hours (left and middle panel). Non-targeted Bcl-2 proteins were analyzed at the same time point by immunoblotting (right panel). (C) Cell death of fibroblasts was determined by quantification of Annexin V (AN)- and propidium iodide (PI)-positive cells 72 hours after transfection of the indicated siRNAs. (D) Cell death analysis of 1205Lu melanoma cells treated as described in C. Asterisks indicate a significant increase in cell death versus control siRNA-treated cells. (E) Fluorescence-activated cell sorting (FACS) of apoptotic and dead fibroblasts (upper panel) or 1205Lu melanoma cells (lower panel) treated as described in C. Numbers indicate the portion of cells in each quadrant that defines Annexin V- or propidium iodide-positive or negative cells. Mean +/− SD of at least 3 independent experiments is shown in B, C, and D. α-tubulin served as loading control in immunoblots. Blots are representative for 3 independent experiments.

Next, the relevance of antiapoptotic Bcl-2 proteins for melanoma cell survival was assessed by silencing them utilizing RNA interference. Effects caused by silencing of Bcl-2, Bcl-xL, Bcl-w, Mcl-1, or A1 were analyzed in the metastatic melanoma cell line 1205Lu. In addition, non-malignant cells were analyzed in order to dissect Bcl-2 proteins that are specifically important for melanoma survival. For this, primary human fibroblasts instead of melanocytes were used as a non-malignant reference. Fibroblasts represent a more prevalent cell type and, in a therapeutic perspective, unspecific cell death is expected to be more serious in ubiquitous cell types than in melanocytes. Furthermore, transfection efficacy of siRNAs is insufficient in melanocytes resulting in inefficient inhibition. ShRNA expression is applicable in melanocytes, e.g., by utilizing lentiviral shRNA-encoding constructs, however, in general, we observe lower silencing efficacies of shRNAs compared to siRNAs. Therefore, it is unlikely to generate the same inhibition efficacies in melanocytes as in melanoma cells. In fibroblasts and melanoma cells, the applied siRNAs efficiently abrogated expression of the respective Bcl-2 molecule (Figure 2A and B). The effect of targeting antiapoptotic Bcl-2 proteins on cell death was analyzed 3 days after siRNA transfection by staining with Annexin V and propidium iodide. In primary fibroblasts, none of the targeted Bcl-2 proteins led to induction of cell death at this time point (Figure 2C and E; upper panel). In melanoma cells, however, significant apoptosis induction was observed when Mcl-1 or A1 were inhibited: Cell death increased from 8% in control siRNA-treated cells to 40% in Mcl-1-inhibited or 30% in A1-inhibited cells (Figure 2D and E; lower panel). A slight increase in cell death was also observed upon targeting of Bcl-2, Bcl-xL, or Bcl-w. Because 3 melanoma cell lines expressed increased amounts of Bcl-xL compared to melanocytes (Figure 1C), we tested whether these cells may be dependent on Bcl-xL. However inhibition of Bcl-xL affected cell death only slightly in WM1232, a cell line with high Bcl-xL levels, (data not shown).

Together, these data show that silencing of individual Bcl-2 proteins does not induce cell death in non-malignant fibroblasts. In contrast, melanoma cells were found to be dependent on the presence of specific antiapoptotic proteins: Inhibition of Mcl-1 or A1 caused significant cell death without further apoptotic stimulus.

### Co-inhibition of Mcl-1 and A1 results in efficient cell death induction of melanoma cell lines without affecting primary human skin cells

**Figure 3:**
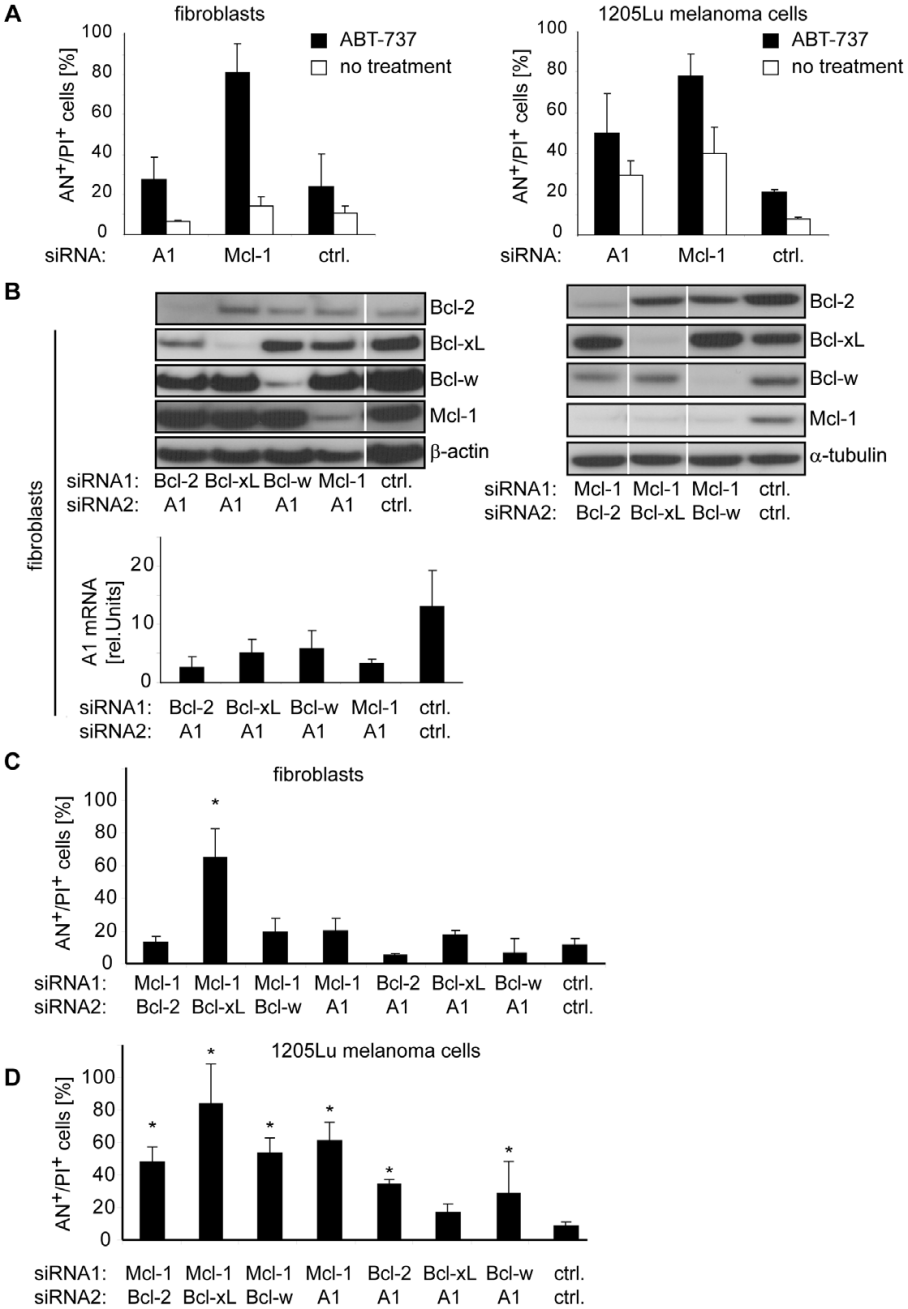
Melanoma-specific cell death can be enhanced by combined inhibition of Mcl-1 and A1. (A) Fibroblasts (left panel) and 1205Lu melanoma cells (right panel) were transfected with the indicated siRNAs for 48 hours. Thereafter, 1 µM ABT-737 was added and cell death was assessed 24 hours after ABT-737 treatment. (B) Fibroblasts were simultaneously transfected with two siRNAs as indicated. 48 hours after transfection the respective protein was analyzed by immunoblotting (upper panels) or RT-PCR (lower panel). Blots are representative for 3 (upper left panel) or 2 (upper right panel) independent experiments. Cell death analysis of (C) fibroblasts or (D) 1205Lu melanoma cells treated with the indicated siRNAs for 72 hours. Mean +/− SD of 3 independent experiments is shown in A, B, C and D. α-tubulin or β-actin served as loading control in immunoblots. White lines indicate lanes that were run at the same blot but are not contiguous. Asterisks represent significant increase in cell death compared to control siRNA-treated cells. AN, Annexin V; PI, propidium iodide.

The results suggested that targeting of the appropriate Bcl-2 proteins, i.e., Mcl-1 or A1, represents a strategy to specifically induce apoptosis in melanoma. A consistent, but not significant increase was also observed when Bcl-2, Bcl-xL, and Bcl-w were targeted in 1205Lu melanoma cells (Figure 2D). We, therefore, tested whether cell death can be enhanced in a tumor-specific manner by targeting additional antiapoptotic Bcl-2 proteins. For this, the BH3 mimetic ABT-737 was used, which inactivates Bcl-2, Bcl-xL, and Bcl-w [13]. In the presence of A1 inhibition, ABT-737 increased cell death to 50% and in the presence of Mcl-1 inhibition to 80%, respectively (Figure 3A; right panel). However in non-malignant fibroblasts, a similar increase of cell death was observed, indicating that simultaneous inactivation of Bcl-2, Bcl-xL, Bcl-w, together with Mcl-1 leads to apoptosis in several cell types (Figure 3A; left panel). As targeting of several antiapoptotic proteins simultaneously was found to enhance apoptosis, we tested whether inhibition of one antiapoptotic Bcl-2 protein together with either Mcl-1 or A1 is able to enhance apoptosis induction in a tumor-specific manner. Using two siRNAs simultaneously, Bcl-2, Bcl-xL, or Bcl-w was individually silenced together with Mcl-1 or A1. In addition, co-inhibition of Mcl-1 and A1 was investigated. In the presence of siRNA-mediated inhibition of Mcl-1 or A1, co-transfection of Bcl-2-, Bcl-xL-, or Bcl-w-specific siRNA resulted in inhibition of the respective two molecules (Figure 3B).

Next, cell death was analyzed. Non-malignant fibroblasts survived the simultaneous inhibition of A1 and Bcl-2, Bcl-xL, or Bcl-w, respectively. Similarly, these cells were not affected by inhibition of Mcl-1 together with Bcl-2 or Bcl-w, respectively, while co-inhibition of Mcl-1 and Bcl-xL strongly induced cell death (Figure 3C). In 1205Lu melanoma cells, cell death induction was observed throughout most combinations, which was expected since inhibition of Mcl-1 or A1 alone already resulted in cell death (Figure 3D). As described in Material and Methods, transfected siRNA amounts in single inhibition experiments were equal to the one in double inhibition experiments allowing the comparison of single versus double inhibition. Cell death in response to Mcl-1 inhibition (around 40% as seen in Figure 2D) was enhanced by additional targeting of Bcl-2, Bcl-xL, Bcl-w, and A1 (50–80%, Figure 3D), respectively. Most strikingly, cell death was induced by Bcl-xL/Mcl-1 co-inhibition, however this was also observed in non-malignant cells (Figure 3C and D) indicating that this combination does not allow cell death induction in a tumor-specific manner.

**Figure 4:**
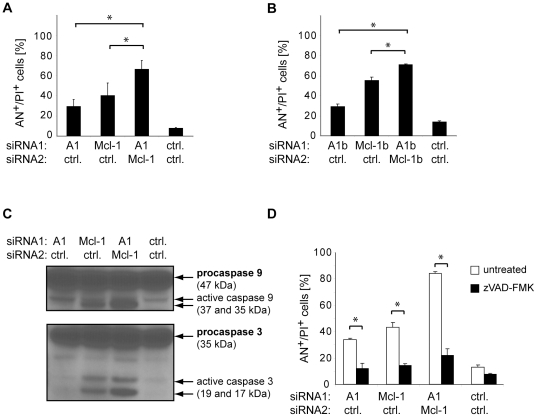
Combined inhibition of Mcl-1 and A1 results in efficient induction of apoptosis in 1205Lu melanoma cells. (A) 1205Lu melanoma cells were transfected with A1- or Mcl-1-specific siRNAs or control siRNAs as indicated. Cell death was assessed 72 hours after transfection. Mean +/− SD of 3 independent experiments is shown. Asterisks represent significant increase in cell death compared to single inhibition. (B) 1205Lu melanoma cells were transfected with different siRNA sequences targeting A1 or Mcl-1 (A1b, Mcl-1b). Analysis was carried out as described in A. Asterisks represent significant increase in cell death compared to single inhibition. Also, cell death induction by A1b, Mcl-1b, and A1b/Mcl-1b versus control was significant (not indicated in the figure). (C) Caspase activation of 1205Lu cells treated as described in A was assessed by immunoblotting with antibodies detecting the proform as well as the active subunits. Caspase 9 (upper panel) and caspase 3 (lower panel) were analyzed 48 hours after transfection. (D) 1205Lu cells treated with siRNAs as described in A with or without the pan-caspase inhibitor zVAD-FMK (100 µM). Cell death was measured 72 hours after transfection. Mean +/− SD of 3 independent experiments is shown. Asterisks represent significant decrease in cell death of zVAD-FMK-treated cells compared to cells not treated with zVAD-FMK. AN, Annexin V; PI, propidium iodide.

Among the tumor-specific combinations, simultaneous inhibition of Mcl-1 and A1 most efficiently induced cell death in 1205Lu melanoma cells. Without further stimulus, around 60% of melanoma cells died whereas fibroblasts remained healthy (Figure 3C and D). The combined inhibition of Mcl-1 and A1 was significantly more effective compared to the single inhibition of the respective gene (Figure 4A). To reduce the probability of siRNA off-target effects, different siRNA sequences targeting Mcl-1 and A1 were examined. Cell death was induced in 1205Lu cells in a similar manner (Figure 4B). To confirm apoptosis as the key mechanism of cell death, caspase activation was analyzed. Immunoblots demonstrated activation of the effector caspase 3 and of caspase 9, a key initiator caspase of the mitochondrial apoptosis pathway (Figure 4C). To functionally test whether caspases are required for cell death, the pan-caspase inhibitor zVAD-FMK was applied. zVAD-FMK almost abolished cell death, indicating the crucial role of caspases for cell death induced by Mcl-1/A1 inhibition (Figure 4D).

**Figure 5:**
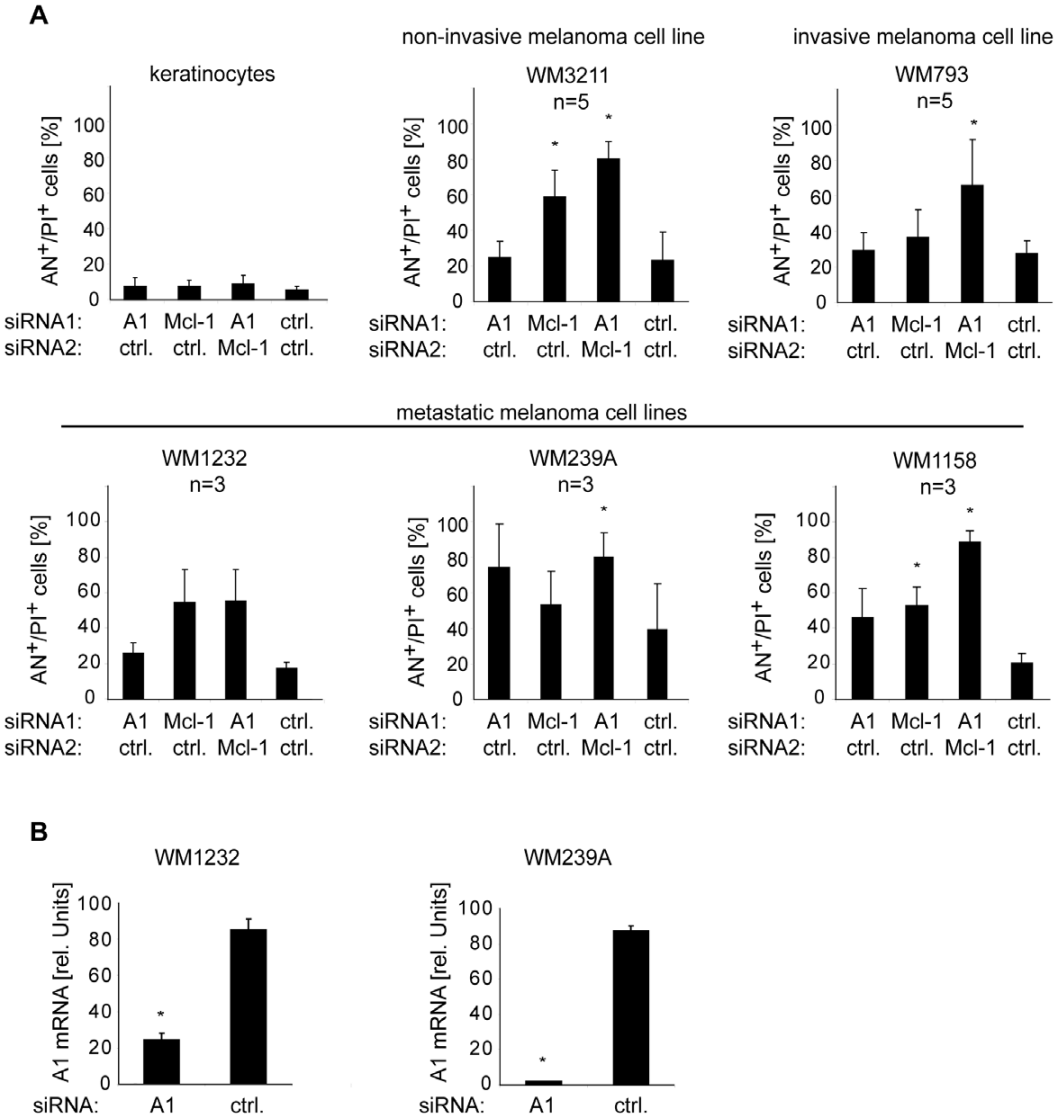
Combined inhibition of Mcl-1 and A1 results in efficient induction of cell death in a panel of melanoma cell lines, but not in keratinocytes. (A) Primary human keratinocytes (upper left panel), non-invasive melanoma cells (WM3211), invasive (WM793), and metastatic melanoma cells (WM1232, WM239A, WM1158) were transfected with the siRNAs as indicated. Cell death was determined 72 hours after transfection. Mean +/− SD of 3 (keratinocytes, WM1232, WM239A, WM1158) or 5 (WM3211 and WM793) independent experiments is shown. (B) A1 mRNA was measured by quantitative RT-PCR in indicated cells treated with A1-specific- or control siRNA for 48 hours. Mean +/− SD of 3 independent experiments is shown. Asterisks represent significant increase in cell death compared to control siRNA-treated cells. AN, Annexin V; PI, propidium iodide.

Next, these analyses were extended to other melanoma cell lines derived from different tumor stages and to primary human keratinocytes, representative for another non-malignant skin cell type. Similar to fibroblasts, keratinocytes were not sensitive to the inhibition of Mcl-1 or A1, nor to the inhibition of both proteins simultaneously (Figure 5A, upper left panel). In melanoma cell lines, substantial sensitivity to Mcl-1 inhibition was observed in 3 of 5 melanoma cells, i.e., WM3211, WM1232, and WM1158 (Figure 5A, other panels). Sensitivity to A1 inhibition was observed in 2 of 5 melanoma cell lines, i.e., WM239A, and WM1158. WM1232 cells were found not to be sensitive to A1 inhibition despite high A1 levels. This may be due to the lower siRNA knock-down efficacy observed in this cell line compared to WM239A, another cell line with high A1 level that was sensitive (Figure 5B). However, the combined inhibition of Mcl-1 and A1 resulted in substantial cell death induction in all melanoma cell lines with rates ranging from 60% to 80%.

Together, the results show that the combined inhibition of Mcl-1 and A1 efficiently induces apoptosis in melanoma cell lines, whereas primary cells remain unaffected.

### Inhibition of Mcl-1 and A1 cooperates with chemotherapy to induce cell death in 1205Lu melanoma cells

**Figure 6:**
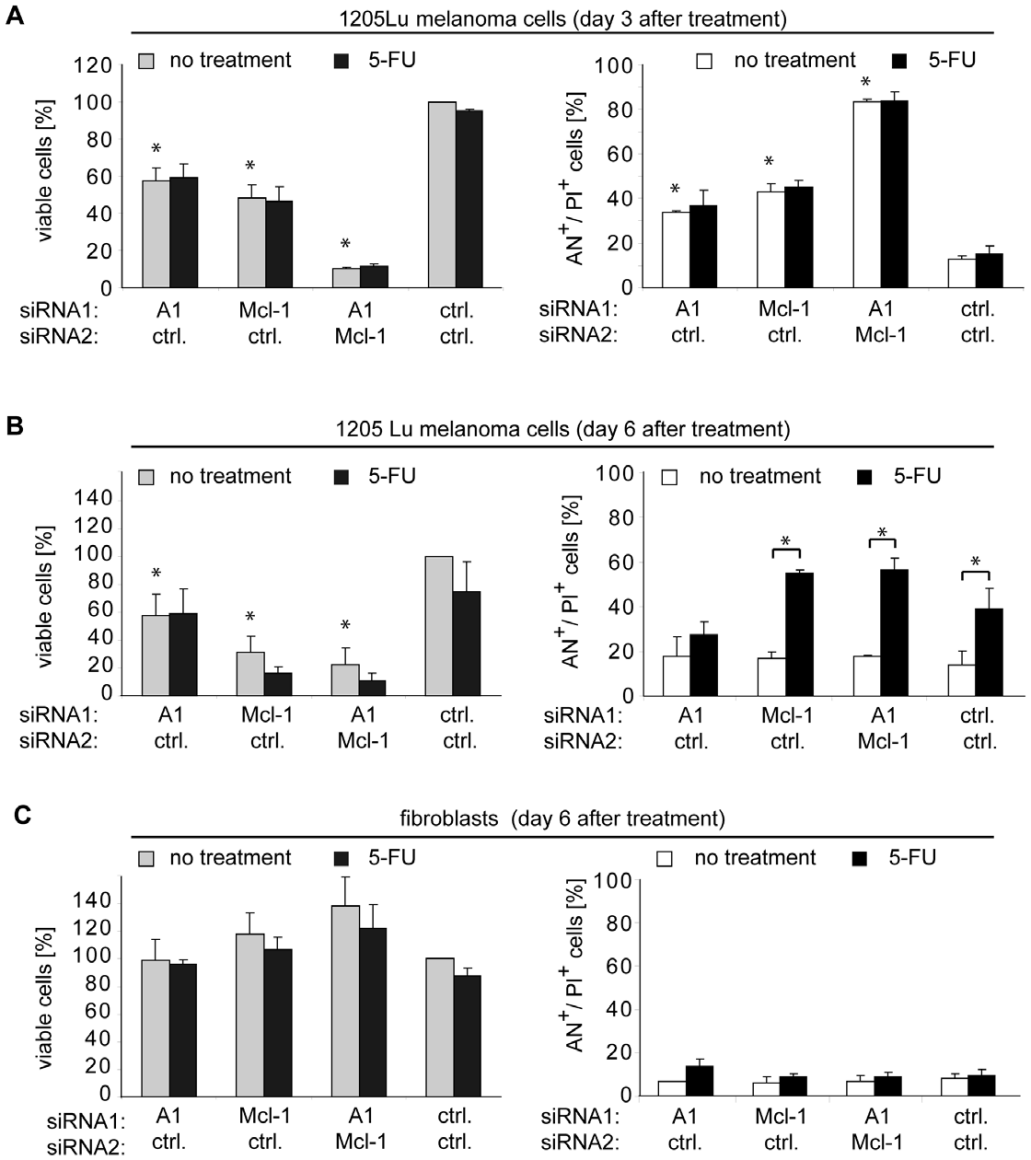
Chemotherapy adds to cell death induced by Mcl-1 and A1 inhibition. (A) 1205Lu melanoma cells were treated with the indicated siRNAs together with 1 µM 5-fluorouracil (5-FU) or not (no treatment) and analyzed on day 3 after transfection. Left panel: Cell viability was determined. Viability of control siRNA-treated cells without 5-FU treatment was set to 100%. Right panel: Cell death analysis measured by FACS after staining with Annexin V and propidium iodide. (B) Analysis of cell viability (left panel) or cell death (right panel) of 1205Lu cells on day 6 treated as described in A. (C) Analysis of cell viability (left panel) or cell death (right panel) of primary human fibroblasts on day 6 treated as described in A. If not otherwise indicated, asterisks represent significant increase in cell death compared to control siRNA-treated cells. Mean +/− SD of 3 independent experiments is shown in A, B, and C. AN, Annexin V; PI, propidium iodide.

**Figure 7:**
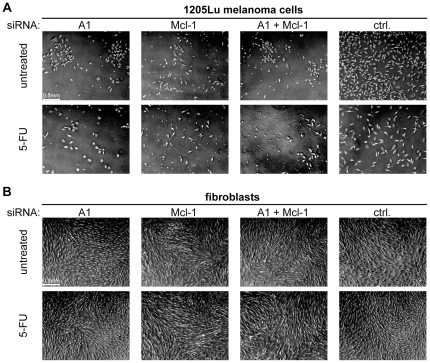
Representative light microscopy images of 1205Lu melanoma cells (A) or fibroblasts (B) 6 days after transfection of the indicated siRNAs alone (upper panel) or together with 1 µM 5-FU (lower panel). Scale bar = 0.5 mm.

We next examined whether cell death induced by inhibition of A1, Mcl-1, or both, can further be enhanced by exposure to chemotherapy, i.e., 5-fluorouracil (5-FU), while maintaining the tumor specificity. Cells were treated with siRNAs with or without 5-FU. Three and 6 days after treatment cell viability was measured by a fluorimetric assay and cell death induction was measured by FACS analysis. On day 3, melanoma cell viability was decreased by inhibition of A1 and Mcl-1, respectively, and – most prominently – by inhibition of both (Figure 6A, left panel). This was associated with induction of cell death as observed before (Figure 6A, right panel). Treatment with 5-FU did not affect cell viability or cell death at this time point. Next cells were analyzed at a later time point on day 6 (Figure 6B). On day 6, 5-FU slightly decreased melanoma cell viability and caused cell death in control samples. A1 or Mcl-1 inhibition, however, reduced cell viability to 60% or 30%, respectively, at this time point, whereas inhibition of both resulted in around 20% surviving cells. In case of treatment with 5-FU, cell viability of Mcl-1-inhibited cells was further reduced from 30% to around 15% and cell viability of Mcl-1/A1-inhibited cells from 20% to 10% (Figure 6B; left panel). Surprisingly when cell death was analyzed in the remaining population by FACS, no apoptotic or dead cells were observed on day 6 in A1/Mcl1-inhibited cells without 5-FU despite a strong reduction in cell viability (Figure 6B; right panel). This suggests that a small portion of cells seems to be unaffected by A1- or Mcl-1-targeting siRNA treatment. However when 5-FU was applied, cell death was significantly increased in this surviving population (Figure 6B; right panel), ultimately leading to the reduction in total cell viability to 10% in Mcl-1/A1-inhibited cells (Figure 6B; left panel). In contrast to 1205Lu melanoma cells, in primary fibroblasts no reduction of cell viability (Figure 6C; left panel) and no increase in cell death (Figure 6C; right panel) was observed on day 6, neither by inhibition of A1, Mcl-1, or A1/Mcl-1 alone nor by co-administration of 5-FU. These results were confirmed morphologically by microscopic evaluation (Figure 7).

Together, the data suggest that chemotherapy cooperates with Mcl-1 and A1 by killing cells that survive A1 or Mcl-1 inhibition. This results in increased cell death while tumor specificity was maintained.

## Discussion

Several studies have analyzed the levels of antiapoptotic Bcl-2 proteins in melanoma, however the results were conflicting and the relevance of Bcl-2 molecules is still unclear [14]. In this study, we aimed at characterizing the relevance of antiapoptotic Bcl-2 proteins in melanoma cell lines in a systematic manner. In previous studies small peptides mimicking certain BH3-only proteins [15] or small molecule Bcl-2 inhibitors have been used [16]. Here, we made use of RNA interference, which enables targeting of all Bcl-2 molecules using the same method thereby allowing better comparison. In addition, the method allows controlling efficacy and targeting specificity by measuring protein or transcript abundance. With this technology we achieved efficient silencing of Bcl-2, Bcl-xL, Bcl-w, Mcl-1, and A1 without affecting the expression of non-targeted antiapoptotic Bcl-2 members. A comparative analysis of the relevance of these molecules was performed not only in melanoma cell lines, but also in non-malignant cells. In a therapeutic perspective, it is very important to specifically target cancer cells while sparing normal cells to avoid unwanted toxicity. However, whereas in many studies antiapoptotic Bcl-2 proteins or BH3 mimetics have been investigated in several cancer types, comparative studies with non-malignant cells to evaluate tumor-specific effects are surprisingly rare. Melanocytes are the non-malignant cell type, melanoma derives from. However, melanocytes represent a rare and specialized cell type. In a therapeutic perspective, the targeting of melanocytes should not harm significantly an organism as opposed to damaging other more prevalent cells with essential physiological functions. Therefore we considered primary fibroblasts, which represent an ubiquitous cell type, and keratinocytes, which represent a mayor cell type of the skin, as a more appropriate non-malignant reference. Furthermore, targeting of melanocytes with RNAi-inducing agents is associated with serious technical problems due to low knock-down efficacies, which complicate the comparison with melanoma cells.

In this systematic screen, Mcl-1, but also A1, have been identified as potential targets whose loss led to strong cell death induction in melanoma cell lines, whereas non-malignant cells remained unaffected. Inhibition of Mcl-1 effectively induced apoptosis in most melanoma cell lines. Newly, A1 was identified as a molecule that is of relevance for melanoma cell survival. Despite less efficient apoptosis induction compared to Mcl-1, combined targeting of Mcl-1 and A1 caused a substantial increase in cell death in the majority of melanoma cell lines tested, without inducing apoptosis in fibroblasts or keratinocytes. Nevertheless, melanoma cell lines were identified that were primarily dependent on Mcl-1 (WM1232) or A1 (WM239A) and the combined treatment did not show an additional benefit. However, all melanoma cell lines tested in our study were sensitive to at least one of both Bcl-2 members. This demonstrates that - even in the presence of varying expression levels and sensitivities among melanoma cell lines - targeting of A1 at least broadens the apoptotic sensitivity of melanoma cells to Mcl-1 inhibition. In a therapeutic perspective, this may be important for the development of multi-specific BH3 mimetics. The intrinsic sensitivity of melanoma cells to Mcl-1 and A1 loss is also supported by several studies that observed melanoma cell death that was associated with induction of the BH3-only protein Noxa [17]–[19]. Noxa specifically inactivates Mcl-1 and A1 [20].

In contrast to melanoma, inhibition of single antiapoptotic proteins did not lead to cell death in non-malignant fibroblasts. However, when targeting two antiapoptotic Bcl-2 proteins simultaneously, the approach to identify tumor-specific combinations turned out to be very important: Inhibition of Mcl-1 in combination with the inhibitor ABT-737 strongly induced cell death, which was also observed by others [21]. However, this seems not to be restricted to tumor cells as the same treatment induced apoptosis in fibroblasts. Similar results were also observed upon inhibition of Mcl-1 together with Bcl-xL. This indicates that certain combinations, despite highly efficient cell death induction, are not advantageous for therapy because they address several cell types leading to general toxicity. Consequently, detailed knowledge of the sensitivity of the targeted cancer type with respect to non-malignant tissue is crucial for the development and for administration of BH3 mimetics that typically target more than one Bcl-2 molecule.

Other studies reported that melanoma cells require Mcl-1 for survival under certain stress conditions, like endoplasmatic reticulum stress, lack of cell adhesion, or proapoptotic stimuli that target the death receptor-mediated apoptotic pathway [22]–[24]. Interestingly, in our study inhibition of Mcl-1 and A1 efficiently induced cell death in a panel of melanoma cell lines without additional stimulus. An intrinsic sensitivity of melanoma cell lines to Mcl-1 inhibition has been observed by others [24]. This indicates that – in contrast to non-malignant cells - melanoma cells require the presence of Mcl-1 or A1 for survival. A potential reason for this sensitivity could be tumor cell-specific alterations: For tumor formation, several cellular properties are necessary which can be acquired by genetic, epigenetic, or other modifications [25]. However, such alterations occur randomly and are associated with other alterations that do not necessarily provide an advantage to the tumor cell. When analyzing antiapoptotic Bcl-2 proteins in non-malignant cells, a melanocyte-specific expression pattern was observed that was consistent within 5 donors. In line with random alterations, this expression pattern was dramatically altered in melanoma and strongly varied between the different cell lines. Gains but also losses in expression levels were observed and the expression profile did not follow a strict pro-survival rule with consistent increase in antiapoptotic molecules. As observed in our study, melanocytes express high levels of Bcl-2 compared to other cell types of the skin [26] and no increase in Bcl-2 expression was observed when melanocytes were compared with melanoma cells [27]. In addition, loss of Bcl-2 expression in melanoma compared to benign nevi has been observed by others [28]. On the other side, random alterations can cause abnormalities that lead to cellular stress, which may render tumor cells sensitive, makes them dependent on certain proteins, and allows them to survive only under specific conditions. This altered dependency has been termed as synthetic lethality or oncogene addiction [10], [11]. Our data argue for a cellular state in which the survival proteins Mcl-1 and A1 are required to balance such a tumor-associated stress in melanoma, and in which their loss leads to cell death without additional apoptotic stimulus.

Chemotherapy represents an apoptotic stimulus that activates apoptosis via multiple pathways and, therefore, may function independently of the more focused effects of Mcl-1 and A1 inhibition. Despite high efficacy in cell death induction by Mcl-1 and A1 inhibition, we observed a small subpopulation of surviving cells (around 20%). The chemotherapeutic agent 5-fluorouracil (5-FU) resulted in significant cell death in this surviving subpopulation. 5-FU reduces DNA synthesis by inhibiting thymidylate synthase, causes DNA damage, and promotes apoptosis via the DNA damage pathway [29]. This indicates that chemotherapy adds to the effect caused by inhibition of Bcl-2 proteins by targeting other apoptosis-inducing pathways.

Together, this study demonstrates that it is possible to efficiently induce cell death specifically in melanoma cells by targeting certain antiapoptotic Bcl-2 proteins. Targeting of more than one Bcl-2 molecule clearly increased efficacy and broadened sensitivity among cell lines without loosing tumor specificity. Further increase can be achieved by the combination with chemotherapy, a treatment that kills 90% of melanoma cell lines without affecting non-malignant fibroblasts. These data provide a basis for future therapeutic approaches based on targeting of Bcl-2 proteins, e.g, by BH3 mimetics. They further underscore the importance to address each Bcl-2 molecule specifically to ascertain that cell death or drug sensitization is restricted to the tumor.

## Materials and Methods

### Reagents and antibodies

Anti–Bcl-2 (Ab-1) antibody was purchased from Merck Biosciences (Darmstadt, Germany). Anti–Bcl-xL, anti–Bcl-w, anti-caspase 3, anti-caspase 9, and HRP-conjugated secondary antibodies were obtained from New England Biolabs (Berlin, Germany). Anti-Mcl-1 (22) antibody was purchased from BD Bioscience (Heidelberg, Germany). Anti–β-actin (AC-15) and Anti-α-tubulin (DM1A) antibodies were obtained from Sigma-Aldrich (Munich, Germany). PCR primers and siRNAs were purchased from Eurofins MWG Operon (Ebersberg, Germany). ABT-737 was purchased from Abbott Laboratories (Abbott Park, Illinois). zVAD-FMK was from Bachem (Bubendorf, Switzerland).

### siRNAs

siRNAs were designed as described previously [30]. Sequences of specific siRNAs were A1: GGAAGAAUUGUAACCAUAU, A1b: CGGAUGUGGAUACCUAUAA, Bcl-2: GGGAGAUAGUGAUGAAGUA, Bcl-w: GGCAGACUUUGUAGGUUAU; Bcl-xL: GGAACUCUAUGGGAACAAU; Mcl-1: GGCAGUCGCUGGAGAUUAU; and Mcl-1b: CCAUGUAGAGGACCUAGAA. Depicted is the 19nt portion corresponding to the sense strand of the targeted mRNA. The sequence of the non-silencing control siRNA (ctrl) was GCGCAUUCCAGCUUACGUA.

### Cell culture

Human melanoma cell lines were a gift of M. Herlyn (Wistar Institute, Philadelphia, Pennsylvania). All were isolated from clinically and histologically defined lesions. Keratinocytes, melanocytes and fibroblasts were isolated from neonatal human foreskins. Cultivation of cells was described previously [17].

### Transfection procedures

Transfections were performed in 6-well dishes in a volume of 1 ml. To enable direct comparison of single and co-inhibition experiments, equal amounts of siRNAs (40 nM) were transfected. In single inhibition experiments, 20 nM of specific siRNA was transfected along with 20 nM control siRNA utilizing 1.6 µl Lipofectamine RNAiMAX (Invitrogen, Darmstadt, Germany) according to the manufacturer's protocol. In co-inhibition experiments, 20 nM of each specific siRNA was used.

### Quantification of viable cells

Viable cells were quantified in 6-well dishes by using a fluorometric assay (CellTiter-Blue Cell Viability Assay; Promega, Mannheim, Germany). Viable cells with intact metabolism were identified by their ability to reduce cell-permeable resazurin to fluorescent resorufin. Medium was replaced with 750 µl of culture medium and 150 µl of CellTiter-Blue reagent. After 1 hour incubation at 37°C, fluorescence was measured.

### Quantification of apoptotic cells and cell death

Adherent and supernatant cells were analyzed by staining with FITC-labeled Annexin V (Roche, Mannheim, Germany) and propidium iodide (Sigma-Aldrich) as described previously [17]. Cells were analyzed by fluorescence-activated cell sorting (FACS) analysis using CellQuest software (BD Bioscience).

### RNA extraction and quantification

Total RNA was extracted from cells using QIAzol Lysis Reagent (Qiagen, Hilden, Germany) as described by the manufacturer and analyzed by quantitative RT-PCR. Reverse transcription and quantification procedures using the LightCycler TaqMan Master Kit (Roche) together with the Universal Probe Library system (Roche) were described previously [17]. Relative gene expression was expressed as a ratio of the expression level of the gene of interest to that of hypoxanthine phosphoribosyltransferase (HPRT) RNA determined in the same sample.

### Protein preparation and immunoblot analysis

Adherent and supernatant cells were lysed in buffer containing 50 mM Tris, pH 7.4, 0.25 M NaCl, 1 mM EDTA, 0.1% Triton X-100, phosphatase inhibitors (PhosSTOP, Roche), and protease inhibitors (Complete, Mini, EDTA-free; Roche). Gel electrophoresis, blotting and protein detection was carried out using the Xcell *SureLock* Mini-Cell apparatus (Invitrogen) as described previously [17]. Protein levels of β-actin or α-tubulin were analyzed as a control for constant loading and transfer.

### Statistics

For statistical analysis, 2-tailed Student's t test was used to assess the significance of mean differences. Differences were considered significant at a P value of 0.05 or less.
